# Immune Cell Infiltration and Identifying Genes of Prognostic Value in the Papillary Renal Cell Carcinoma Microenvironment by Bioinformatics Analysis

**DOI:** 10.1155/2020/5019746

**Published:** 2020-07-25

**Authors:** Ting Liu, Man Zhang, Deming Sun

**Affiliations:** ^1^Department of Nephrology, The People's Hospital of China Three Gorges University; The First People's Hospital of Yichang, Yichang 443000, Hubei, China; ^2^Department of Urology, The People's Hospital of China Three Gorges University; The First People's Hospital of Yichang, Yichang 443000, Hubei, China

## Abstract

Papillary renal cell carcinoma (PRCC) is one of the most common histological subtypes of renal cell carcinoma. Type 1 and type 2 PRCC are reported to be clinically and biologically distinct. However, little is known about immune infiltration and the expression patterns of immune-related genes in these two histologic subtypes, thereby limiting the development of immunotherapy for PRCC. Thus, we analyzed the expression of 22 immune cells in type 1 and type 2 PRCC tissues by combining The Cancer Genome Atlas (TCGA) database with the ESTIMATE and CIBERSORT algorithms. Subsequently, we extracted a list of differentially expressed genes associated with the immune microenvironment. Multichip mRNA microarray data sets for PRCC were downloaded from the Gene Expression Omnibus (GEO) to further validate our findings. We found that the immune scores and stromal scores were associated with overall survival in patients with type 2 PRCC rather than type 1 PRCC. Tumor-infiltrating M1 and M2 macrophages could predict the clinical outcome by reflecting the host's immune capacity against type 2 PRCC. Furthermore, CCL19/CCR7, CXCL12/CXCR4, and CCL20/CCR6 were shown to be potential new targets for tumor gene therapy in type 2 PRCC. Our findings provide valuable resources for improving immunotherapy for PRCC.

## 1. Introduction

Papillary renal cell carcinoma (PRCC) is the second most common histological subtype of renal cell carcinoma after the clear cell subtype, where it accounts for 15% to 20% of kidney cancers [1]. Type 1 and type 2 PRCC are shown to be different types of renal cancer with distinct histologic features and diverse clinical prognosis. Compared with type 1 PRCC, type 2 PRCC is more heterogeneous and aggressive which is also associated with higher mortality. Successful treatment is challenging after metastasis occurs because the targeted drugs are generally less robust [2]. The drug resistance mechanism remains unclear, and there is no standard therapy for metastatic PRCC patients at present. PRCC has attracted increasing attention recently, especially in the field of immunotherapy.

The tumor microenvironment clearly affects the survival and development of tumor cells, and it generally has two main components, where the cellular component is characterized by the infiltration of different cells such as inflammatory cells, immune cells, mesenchymal stem cells, and fibroblast cells, whereas the noncellular component mainly comprises cytokines and chemokines [3, 4]. The components of the tumor microenvironment are highly complex, and each has different effects on cell proliferation, tumor distant metastasis, and drug resistance [5, 6]. In the microenvironment system, stromal and immune cells are two important nontumor components, and they are suitable targets for immunotherapy and prognosis evaluation in various cancers such as melanoma [7], non-small cell lung cancer [8], and prostatic cancer [9]. In addition, previous studies have shown that the tumor microenvironment can have significant impacts on the expression of genes in tumor tissues [10]. Further investigations of genetic changes in specific tumor microenvironments may facilitate targeted therapy.

Two algorithms called the MAlignant Tumor tissue using Expression data (ESTIMATE) and the Cell-type Identification By Estimating Relative Subsets Of known RNA Transcripts (CIBERSORT) were developed recently. The ESTIMATE algorithm was designed by Yoshihara et al., and it can be used for calculating immune and stromal scores by analyzing the specific gene expression characteristics of immune cells and stromal cells and for predicting the infiltration of nontumor cells in tumor tissue [11]. CIBERSORT is a gene expression-based deconvolution algorithm invented by Newman et al. that utilizes a matrix of 547 barcode genes for characterizing immune cell components in order to identify the diversity and full range of infiltrating immune cells [12]. These two useful algorithms have been applied in microenvironment research for various cancers [13–15], thereby demonstrating the practical utility of big data-based arithmetic.

Little is known about the immune cell infiltration profile and genetic basis of PRCC, thereby limiting the development of immune treatments for PRCC. Thus, in the present study, we conducted a comprehensive assessment of tumor microenvironment-related cells and genes, as well as determined the effects of immune signatures on the outcomes of type 1 and type 2 PRCC patients. We analyzed the expression patterns of 22 immune cells in these two tumor entities by combining The Cancer Genome Atlas (TCGA) database with the ESTIMATE and CIBERSORT algorithms. Subsequently, we extracted a list of differentially expressed genes (DEGs) associated with the immune microenvironment. These correlations were validated using multichip mRNA microarray data sets for PRCC downloaded from the Gene Expression Omnibus (GEO).

## 2. Materials and Methods

### 2.1. Data Acquisition

The gene expression profiles and corresponding clinical information were downloaded from the TCGA website for kidney renal papillary cell carcinoma projects (TCGA-KIRP) (available online: https://portal.gdc.cancer.gov/). Immune scores and stromal scores were calculated using the ESTIMATE algorithm (available online: https://bioinformatics.mdanderson.org/estimate/). The CIBERSORT algorithm (available online: https://cibersort.stanford.edu/) was applied to analyze the normalized gene expression matrix and to calculate the relative proportions of 22 infiltrating immune cells. Validation was conducted using expression profiles GSE7023 and GSE2748 downloaded from GEO (Affymetrix Human Genome U133 Plus 2.0 platform) for 12 paracarcinoma tissues and 33 type 1 and 31 type 2 PRCC tissues (available online: https://www.ncbi.nlm.nih.gov/geo/).

### 2.2. Processing and Aggregation of Raw Data

R (version 3.6.0) package “Limma” [16] was used to process and merge data downloaded from TCGA in order to obtain stromal and immune cell scores, as well as the normalized gene expression matrix in the tumor microenvironment. The SVA package [17] with the ComBat function was used to normalize the multichip GEO data set to remove batch effects and obtain a standardized gene expression matrix. Normalized gene expression data from TCGA and GEO were uploaded to the CIBERSORT website and analyzed automatically using the default signature matrix with 1000 permutations. Subsequently, we selected the filtered cases with *P* values < 0.05 for further analysis because CIBERSORT generates a *P* value for the deconvolution of each sample using Monte Carlo sampling [18].

### 2.3. Different Immune Cell Expression Patterns in PRCC and Normal Tissues

The data obtained after filtration were used to analyze the expression patterns of 22 immune cell proportions in PRCC and normal tissues, where *P* < 0.05 indicated a statistically significant difference. Correlation analysis was performed based on 22 immune cells in order to determine the internal relationships among each of the immune cells.

### 2.4. Identification of DEGs in PRCC Immune Microenvironment

The data were rearranged according to high and low immune scores in order to identify DEGs associated with the immune microenvironment. The cutoff criteria were specified as follows: fold change > 1.5 and adjusted *P* value < 0.05.

### 2.5. Functional Enrichment Analysis of DEGs and Screening for Key Modules

The Database for Annotation, Visualization and Integrated Discovery (DAVID) is used widely for biological information analysis (available online: https://david.ncifcrf.gov/). We utilized DAVID to perform Gene Ontology (GO) and Kyoto Encyclopedia of Genes and Genomes (KEGG) pathway enrichment analysis for DEGs. An adjusted *P* < 0.05 indicated a significant difference. In order to identify the key modules among the DEGs, a protein-protein interaction (PPI) network was generated using the Search Tool for the Retrieval of Interacting Genes (STRING) (version 10.5) online database (available online: https://string-db.org/). An interaction score > 0.95 was considered meaningful. Cytoscape software (version 3.7.0) [19] was used to visualize interacting networks of DEGs. Subsequently, we used Molecular Complex Detection (MCODE) (version 1.4.2) to identify key modules in the established network. The screening criteria comprised: max depth = 100, degree cutoff = 2, *k*‐score = 2, MCODE score > 5, and node score cutoff = 0.2.

### 2.6. Prognostic Value of Microenvironment in PRCC

Clinical information was downloaded from TCGA. Patients with any missing data were excluded. Corresponding survival analyses were conducted for PRCC patients using the Kaplan–Meier method. The log-rank test was conducted, and a *P* value < 0.05 indicated a significant difference.

## 3. Results and Discussion

### 3.1. Relationships between Stromal/Immune Scores and Prognosis in PRCC Patients

**Figure 1:**
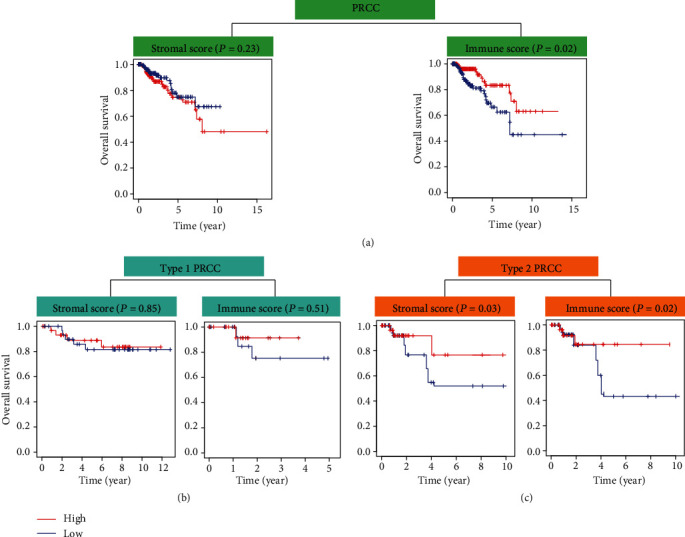
Relationships between stromal/immune scores and prognosis in PRCC patients. (a) Effects of stromal scores and immune scores on overall survival rate in PRCC patients. Effects of stromal/immune scores on overall survival rate in (b) type 1 and (c) type 2 PRCC patients.

**Figure 2:**
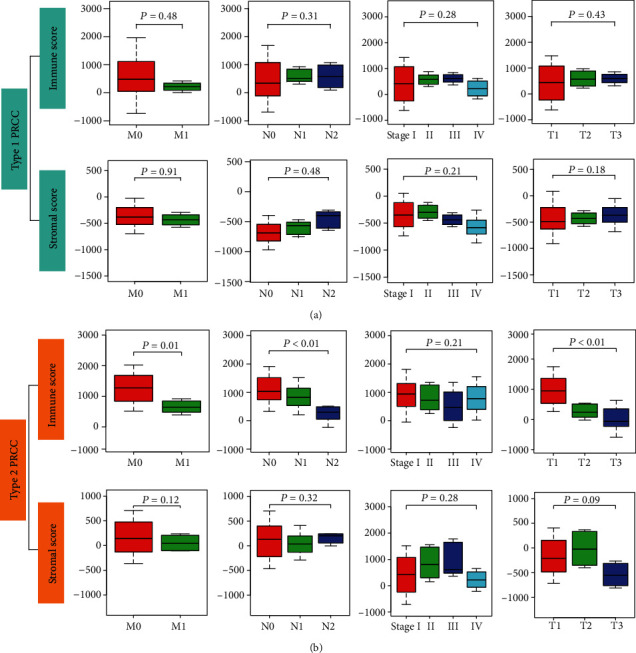
Relationship between stromal/immune scores and clinical characteristics. Associations between stromal/immune scores and distant metastasis, lymph nodes, clinical stage, and topography in (a) type 1 PRCC patients and (b) type 2 PRCC patients. M: distant metastasis; N: lymph node; T: topography.

The gene expression matrix and related clinical information for 136 PRCC tissues were downloaded from TCGA. In order to evaluate the effects of stromal/immune scores on the overall survival rates of PRCC patients, we divided 136 PRCC cases into two groups (high vs. low score groups) according to the stromal or immune scores. Kaplan–Meier survival curves indicated that the stromal score was not related to overall survival, but a low immune score was associated with worse overall survival (Figure 1(a)). Additionally, after dividing tumor tissues into type 1 and type 2 PRCC, the result showed that immune scores and stromal scores were associated with overall survival in patients with type 2 PRCC rather than type 1 PRCC (Figures 1(b) and 1(c)). Wilcoxon's signed-rank test was used to further investigate the effects of the stromal and immune scores on clinical characteristics, such as the tumor stage, distant metastasis, lymph node, and topography. The results indicated that both the stromal and immune scores had no meaningful effects on clinical characteristics in patients with type 1 PRCC (Figure 2(a)). Notably, immune scores rather than stromal scores were associated with tumor stage, distant metastasis, and topography in type 2 tumor entity, indicating that low immune scores reflected an aggressive clinical course in type 2 PRCC patients (Figure 2(b)).

### 3.2. Infiltration by 22 Immune Cell Types in PRCC and Normal Tissues

**Figure 3:**
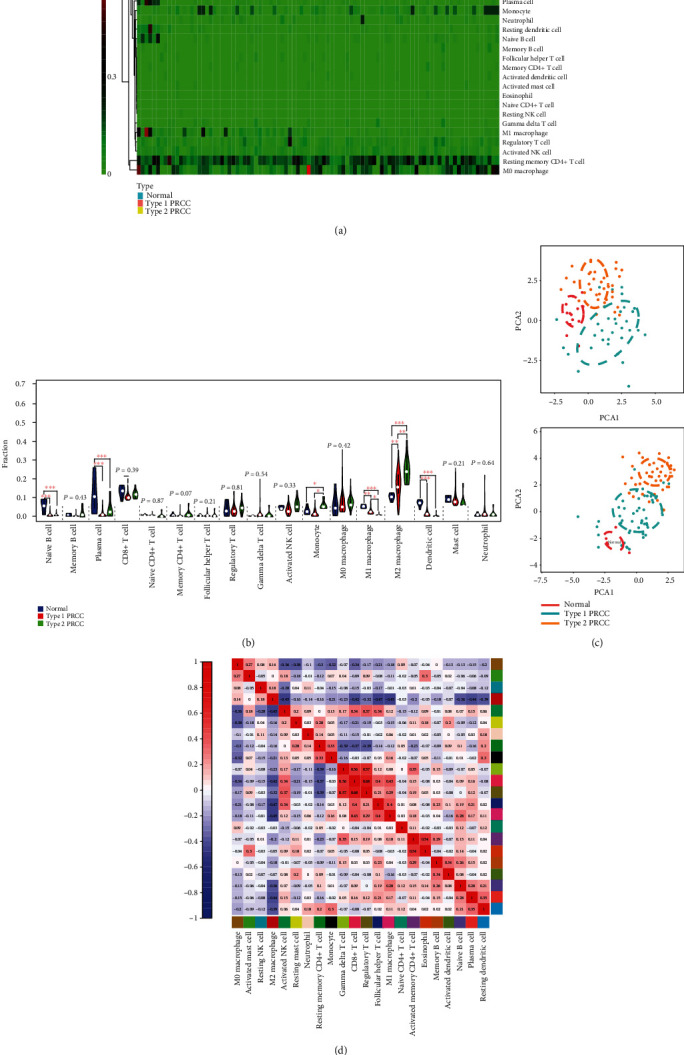
Profiles of infiltrating immune cells in normal and PRCC tissues. (a) Heat map based on proportions of 22 immune cell types in normal, type 1, and type 2 PRCC tissues. (b) Violin plot showing differences in the expression of 22 immune cell types in normal (blue), type 1 (red), and type 2 (green) PRCC groups. (c) PCA analyses indicating group-biased clustering of immune cells from 76 GEO cases and 95 TCGA cases. (d) Correlation analysis based on 22 immune cell subpopulations. ^∗^*P* < 0.05, ^∗∗^*P* < 0.01, and ^∗∗∗^*P* < 0.001.

During the calculation process, CIBERSORT allocates a *P* value for deconvolution to each sample in order to estimate the reliability of each result. Cases with a *P* value < 0.05 were considered more credible. Therefore, the filtered files with *P* values < 0.05 were eligible for further analyses. After screening, we selected 95 TCGA cases (6 normal tissues, 38 type 1 PRCC, and 51 type 2 PRCC tissues) and 76 GEO cases (12 normal tissues, 33 type 1 PRCC, and 31 type 2 PRCC tissues) to investigate the profiles of 22 infiltrating immune cells. The results showed that each type of immune cell exhibited different infiltration properties in type 1 and type 2 PRCC tissues. Compared with normal tissues, PRCC tissues generally contained a lower proportion of M1 macrophages, naive B cells, plasma cells, and dendritic cells, whereas the fractions of the M2 macrophage and monocyte were relatively higher (Figure 3(a)). Importantly, type 2 was characterized by M2 macrophage infiltration and M1 macrophage deficiency which differed significantly from type 1 PRCC (Figure 3(b)), revealing a potential role of M2 and M1 macrophages in PRCC. To validate these preliminary conclusions, we performed the same analysis using GEO data, and the detailed results are presented in Supplementary Figs. S1a and S1b. Surprisingly, the results obtained based on the two databases were almost identical. In fact, the distributions of cells exhibited obvious group-biased clustering according to principal component analysis (PCA) in different tissues (Figure 3(c)), which showed that the gene expression pattern of type 1 PRCC was different from that of type 2 PRCC. Additionally, correlation analysis revealed that the relationships between immune cell subpopulations were low to moderate (Figure 3(d)).

### 3.3. Associations between Key Immune Cell Types and Overall Survival in PRCC Patients

**Figure 4:**
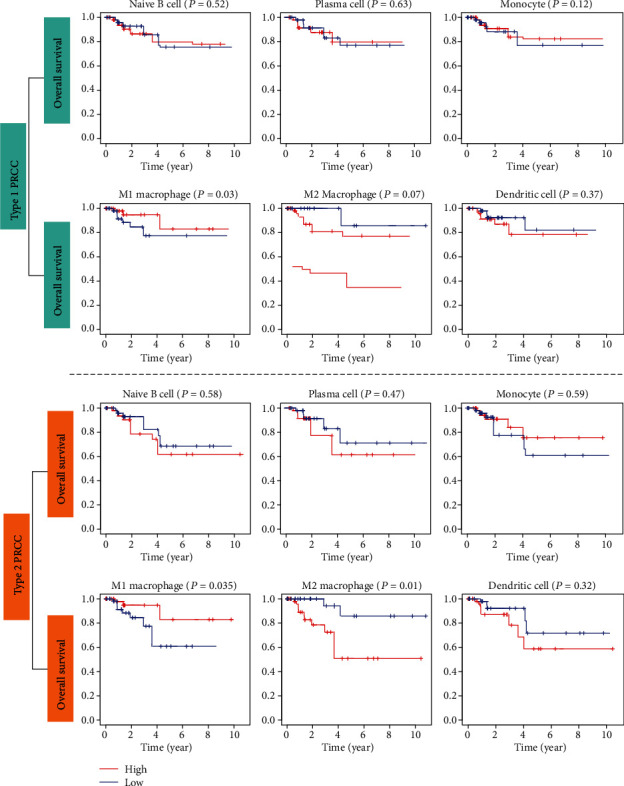
Kaplan–Meier survival curves obtained to investigate the impacts of key immune cell types on overall survival in type 1 and type 2 PRCC patients. A *P* value < 0.05 was considered to indicate a significant difference based on the log-rank test.

Immune cells that differed significantly between PRCC and normal tissues were selected to perform survival analyses. Clinical data for 89 PRCC patients were downloaded from TCGA. The results showed that high proportions of M2 macrophages were associated with a worse survival rate in patients with type 2 PRCC, whereas M1 macrophages had the opposite association. However, no significant associations between immune cell types and overall survival were found in patients with type 1 PRCC (Figure 4).

**Figure 5:**
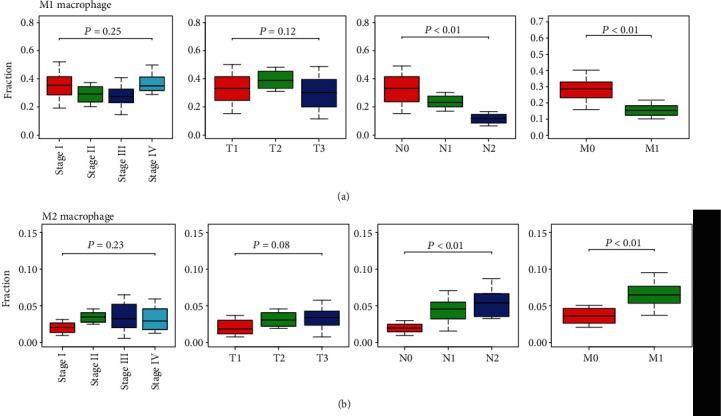
Relationships between survival-related immune cells and clinicopathological characteristics in patients with type 2 PRCC. (a) Associations between M1 macrophages and clinical stage, topography, lymph nodes, and distant metastasis. (b) Associations between M2 macrophages and clinicopathological characteristics. T: topography; N: lymph node; M: distant metastasis.

We further evaluated the relationships among survival-related immune cells and the clinicopathological characteristic in patients with type 2 PRCC. Subgroup analysis indicated that the proportion of M1 macrophages exhibited a decreasing trend with the development of topography and distant metastasis in patients with type 2 PRCC (Figure 5(a)). By contrast, an increasing trend in the M2 macrophage fraction was associated with tumor progression (Figure 5(b)).

### 3.4. Identification of DEGs and Key Genes in PRCC Immune Microenvironment

**Figure 6:**
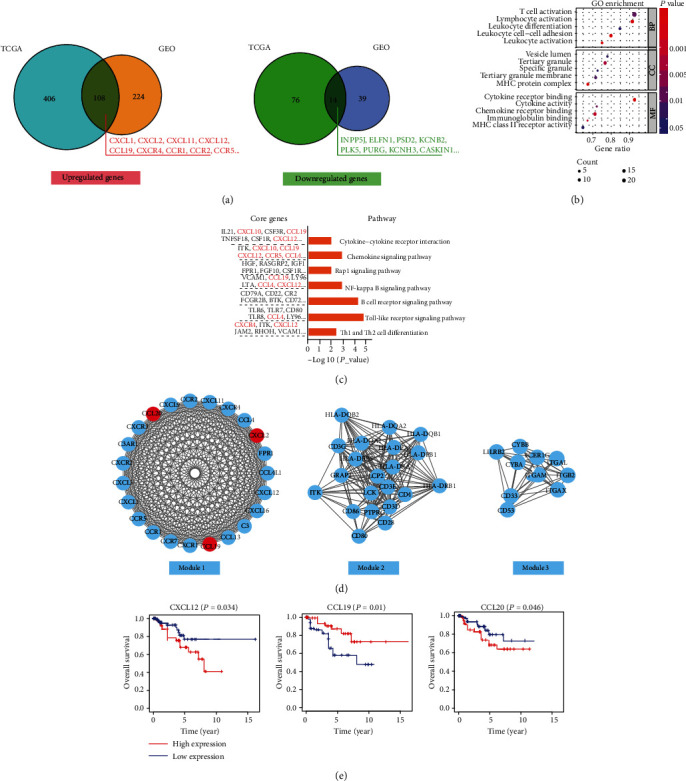
Functional enrichment analysis and selection of key modules for DEGs in type 2 PRCC tissues. (a) Overlapping genes identified in TCGA and GEO databases. Significant (b) GO terms and (c) KEGG pathways for the upregulated genes identified in the high immune score group of type 2 PRCC. (d) Three meaningful modules in the PPI network. (e) Kaplan–Meier curves were prepared according to the high and low expression levels of core genes in type 2 PRCC.

As mentioned above, higher immune scores were related to better overall survival in patients with type 2 PRCC. To explore the different gene expression profiles in the type 2 PRCC immune microenvironment, we ordered the data according to the high and low immune scores. In the high immune score group, 122 overlapping DEGs were identified in TCGA and GEO databases, with 108 significantly upregulated genes and 14 downregulated genes (Figure 6(a)). GO and KEGG enrichment analyses were performed to determine the functions and pathways associated with these DEGs. The results showed that immune cell adhesion or activation and cytokine/chemokine binding function were the most significant biological process and molecular function identified for these genes (Figure 6(b)). In addition, cytokine-cytokine receptor interaction, chemokine signaling pathway, and Toll-like receptor pathway were the top pathways determined by KEGG analysis (Figure 6(c)). Subsequently, we performed PPI network analysis to analyze the potential interplay among the DEGs using the online STRING tool. The PPI network obtained for the DEGs is illustrated in Supplementary Fig. S2. We obtained three meaningful modules using Cytoscape software, where module 1 contained 23 hub genes (e.g., CCL20, CCl19, and CXCL12), and there were 21 hub genes and 10 hub genes in module 2 and module 3 (Figure 6(d)), respectively. These genes were closely related to each other, revealing a complicated regulatory network in type 2 PRCC. Additionally, we prepared Kaplan–Meier survival curves based on 51 type 2 PRCC samples downloaded from the TCGA database to analyze the effects of the hub genes on overall survival. We found that high expression levels of CCL19 were associated with better overall survival in type 2 PRCC patients, whereas CXCL12 and CCL20 showed opposite outcomes (Figure 6(e)).

## 4. Discussion

Emerging evidence indicates that patients in the same TNM stage might exhibit differences in their overall survival because the TNM staging system focuses only on the traits of the tumor itself, and it fails to fully consider the immune characteristics of tumor tissues [20]. The purity of a tumor and infiltrating immune cells may have crucial effects on the clinical outcomes of patients with kidney cancer [21, 22], thereby suggesting that the Immunoscore system could complement the prognostic judgment system for tumors. Recently, the application of immunotherapies such as PD-1/PD-L1 inhibitors or tumor vaccines has highlighted the importance of tumor immune environment traits for PRCC [23, 24]. However, the tumor microenvironment for PRCC, especially for the different pathological subtypes, remains poorly understood, and there is a lack of immune biomarkers for predicting the prognosis or therapeutic effect for patients.

This study is the first to determine the relationships between immune/stromal cells and clinical characteristics in PRCC patients. We found that a higher Immunoscore was associated with better overall survival in patients with type 2 PRCC, thereby suggesting that local immune infiltration probably has potential antitumor effects in type 2 PRCC. However, we did not observe this association in type 1 PRCC, revealing that type 1 and type 2 PRCC are distinctly different kidney tumors with specific gene expression patterns. Additionally, type 2 was shown to be a more aggressive PRCC subtype than type 1, and our findings have more important clinical significance for type 2 PRCC. Previous studies have highlighted the important roles of immune microenviroment in tumor metastasis and immunotherapy. High Immunoscore was believed to be associated with a lower number of metastases and a better clinical prognosis in colorectal cancer patients [25]. Advanced urothelial carcinoma patients with high Immunoscores could obtain greater benefits from immunotherapy, where the predictive accuracy of the microenvironmental score was comparable with that of the tumor mutation burden [26]. It seems that tumor tissues with a small amount of immune cell infiltration are more likely to achieve tumor immune escape [27]. Thus, a high Immunoscore can indicate a strong antitumor immune response and a better immunotherapeutic effect.

Immune cell subpopulations can exhibit diverse cellular infiltration patterns in different tumors. Therefore, determining the distributions and amounts of different infiltrating immune cells in tumors and evaluating the function of each cell subset are major priorities [28]. In this study, data from TCGA and GEO were combined to establish the immune cell infiltration profiles in PRCC tissues. Interestingly, we found that M2 macrophages had higher infiltration levels in type 2 PRCC tissues than paracarcinoma and type 1 tissues, and this variation was closely associated with distant metastasis and worse survival, whereas the opposite trend was determined for M1 macrophages. These findings suggested that macrophages might be a promising breakthrough of immunotherapy for type 2 PRCC. In fact, macrophage phenotypes are critical mediators of immune regulation and tumor metastasis [29], but these “polarized states” are debatable for macrophages [30]. The M1 phenotype (antitumoral) and M2 phenotype (protumoral) may simply represent the extremes in a series of macrophage functional states rather than different actual cell subsets [30]. Our results highlight the importance of the functional status of macrophage subpopulations and the potential capacity for using tumor-associated macrophages as biomarkers for type 2 PRCC. It should be noted that multiple immune escape mechanisms exist in tumors to inhibit CD8+ T cell attacks. A high level of CD4+/CD8+ T cells is linked with an extended prognosis in various tumors [31–34], but we failed to establish this correlation in the present study, thereby implying that macrophages might play more important roles in PRCC rather than CD4+/CD8+ cells [35].

High-throughput sequencing allows researchers to obtain a better understanding of the DEGs in tumor microenvironments. Previous studies determined the upregulation of genes related to immune cell activation (e.g., CD3D, CD8, CXCR3, CCL5, CXCL9, and CXCL10) in cancer patients with a better prognosis [36–38], thereby highlighting the impacts of immune-related genes on tumor progression. We screened 108 upregulated DEGs in a high Immunoscore microenvironment in type 2 PRCC tissues. Functional enrichment analysis indicated that these genes are mainly responsible for regulating cell-cell adhesion and cytokine/chemokine signaling pathway activation. Variations in the cell-cell adhesion capacity are an important component of cancer cell invasion and metastasis [39, 40]. In addition, tumor-infiltrating immune cells regulate cytotoxic effects and cancer-related inflammation by secreting various cytokines and chemokines [41]. Thus, the genes we identified are closely related to the development of type 2 PRCC.

The core genes were associated with immune cell activation functions, including inflammation and immunity genes (e.g., CD3D, CD4, CD28, and HLA), chemokine genes (e.g., CCL20, CXCL2, CCL19, and CXCL1), and chemokine relative receptors (e.g., CXCR4, CCR6, and CCR7). It has been suggested that the expression of CD3 and CD8 in the center of tumors and invasive marginal areas can predict postoperative survival [42]. Correlations between immunity genes and overall survival were not found in our study, but we observed that type 2 PRCC patients with high expression levels of chemokine CXCL12 or CCL20 had a low survival rate, whereas the high level expression of CCL19 had antitumor effects. CXCL12 and CCL20 were shown to be stimulatory chemokines related to tumor neovascularization [43]. In addition, the CXCL12/CXCR4/CXCR7 and CCL20/CCR6 chemokine axes are known to promote migration and the invasiveness of cancer [44, 45]. By contrast, CCL19/CCR7 executes antitumor effects via the induction of the Th1-polarized T cell immunity response [46] and an epithelial-to-mesenchymal transition [47]. CCL19 is also an adjuvant for DNA vaccination [46], and it can improve the body's resistance to tumor cells. Therefore, chemokines and their related receptors are important elements that affect clinical outcomes, and different types of chemokines may have diverse effects on type 2 PPRC progression.

## 5. Conclusions

In the present study, we determined the detailed immune landscape for type 1 and type 2 PRCC by elucidating distinct immune infiltration patterns and assessing the prognostic value of different immune-infiltrating cells. We highlighted the important functions and prognostic value of M1 macrophages, M2 macrophages, and chemokines CCL19, CCL20, and CXCL20 in type 2 PRCC. These findings enhance our understanding of immune infiltration and the expression patterns of immune-related genes in PCRR, which may be valuable for improving immunotherapy approaches.

## Data Availability

The data used to obtain our results are available from GEO (https://www.ncbi.nlm.nih.gov/geo/) and TCGA (https://portal.gdc.cancer.gov/), as well as the ESTIMATE (https://bioinformatics.mdanderson.org/estimate/) and CIBERSORT (https://cibersort.stanford.edu/) algorithm websites.

## Supplementary Materials

Supplementary Materials Figure S1: immune cell infiltration profiles determined in normal and PRCC tissues using GEO data. Figure S2: protein-protein interaction network of differentially expressed genes in type 2 PRCC tissues.
