# Supplementary material for: Immune Cell Infiltration and Identifying Genes of Prognostic Value in the Papillary Renal Cell Carcinoma Microenvironment by Bioinformatics Analysis
Source: Biomed Res Int. 2020 Jul 25;2020:5019746. doi: 10.1155/2020/5019746 (PMC7399742; doi:10.1155/2020/5019746)
Supplement: Supplementary Materials — Figure S1: immune cell infiltration profiles determined in normal and PRCC tissues using GEO data. Figure S2: protein-protein interaction network of differentially expressed genes in type 2 PRCC tissues. [file 5019746.f1.pdf]

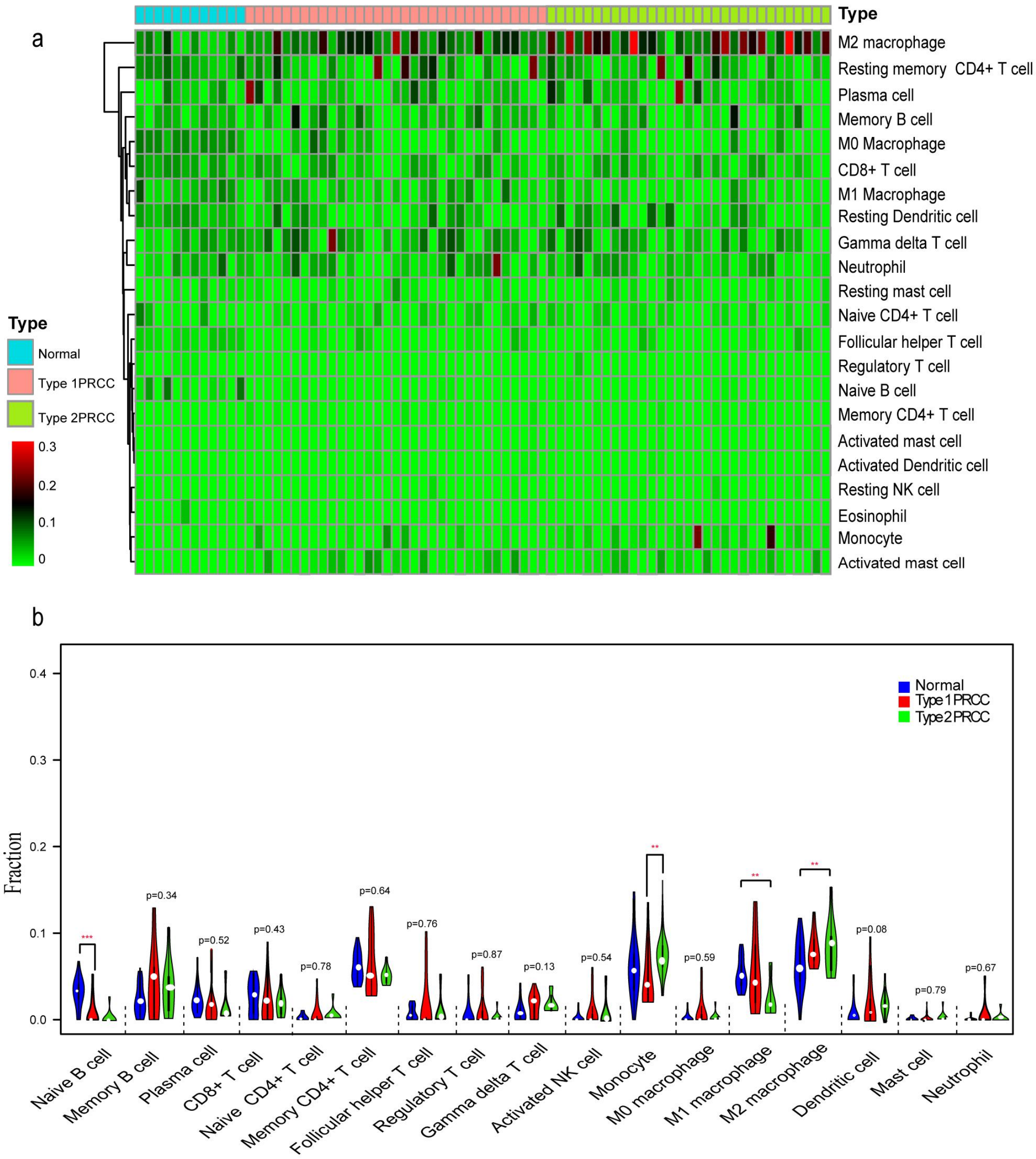

Figure S1. Profiles of infiltrating immune cells in normal and PRCC tissues using GEO data. (a) Heatmap of 22 immune cell proportions in normal tissues, type 1 and type 2 PRCC tissues. (b) Violin plot showed the different expression of 22 immune cells between normal (blue), type 1 (red) and type 2 PRCC groups (green). *P < 0.05, **P < 0.01, ***P < 0.001.

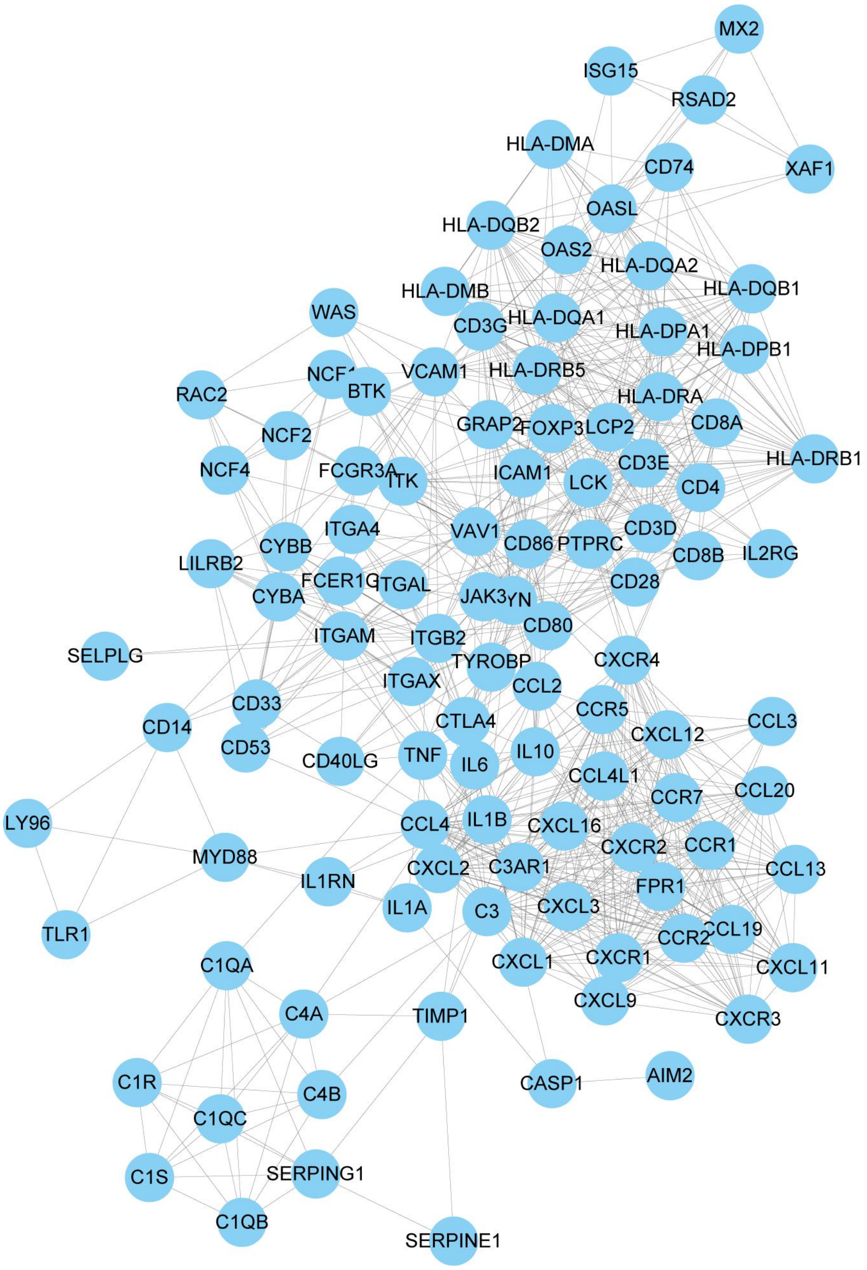

Figure S2. Protein–protein interaction network of differentially expressed genes in type 2 PRCC tissues.
